# Experimental Characterization of Optimized Piezoelectric Energy Harvesters for Wearable Sensor Networks

**DOI:** 10.3390/s21217042

**Published:** 2021-10-24

**Authors:** Petar Gljušćić, Saša Zelenika

**Affiliations:** 1 University of Rijeka, Faculty of Engineering, Vukovarska 58, 51000 Rijeka, Croatia; pgljuscic@riteh.hr; 2 University of Rijeka, Centre for Micro- and Nanosciences and Technologies, Radmile Matejčić 2, 51000 Rijeka, Croatia

**Keywords:** piezoelectric energy harvesters, medical sensor networks, DoE, frequency up-conversion, optimized geometry, FE numerical modelling, experimental assessment

## Abstract

The development of wearable devices and remote sensor networks progressively relies on their increased power autonomy, which can be further expanded by replacing conventional power sources, characterized by limited lifetimes, with energy harvesting systems. Due to its pervasiveness, kinetic energy is considered as one of the most promising energy forms, especially when combined with the simple and scalable piezoelectric approach. The integration of piezoelectric energy harvesters, generally in the form of bimorph cantilevers, with wearable and remote sensors, highlighted a drawback of such a configuration, i.e., their narrow operating bandwidth. In order to overcome this disadvantage while maximizing power outputs, optimized cantilever geometries, developed using the design of experiments approach, are analysed and combined in this work with frequency up-conversion excitation that allows converting random kinetic ambient motion into a periodical excitation of the harvester. The developed optimised designs, all with the same harvesters’ footprint area of 23 × 15 mm, are thoroughly analysed via coupled harmonic and transient numerical analyses, along with the mostly neglected strength analyses. The models are validated experimentally via innovative experimental setups. The thus-proposed *ϕ* = 50 mm watch-like prototype allows, by using a rotating flywheel, the collection of low-frequency (ca. 1 to 3 Hz) human kinetic energy, and the periodic excitation of the optimized harvesters that, oscillating at their eigenfrequencies (~325 to ~930 Hz), display specific power outputs improved by up to 5.5 times, when compared to a conventional rectangular form, with maximal power outputs of up to >130 mW and average power outputs of up to >3 mW. These power levels should amply satisfy the requirements of factual wearable medical systems, while providing also an adaptability to accommodate several diverse sensors. All of this creates the preconditions for the development of novel autonomous wearable devices aimed not only at sensor networks for remote patient monitoring and telemedicine, but, potentially, also for IoT and structural health monitoring.

## 1. Introduction

Innovative wearable devices worn by the users as accessories, e.g., as a watch or a clothing element, are the result of the ongoing development of a new class of electronic gadgets characterized by rapidly decreasing size and power requirements, increasingly used in biomedical applications [1,2]. In order to operate, such devices require a reliable power source. When in the form of a conventional or a rechargeable battery, these sources have, however, limited lifetimes and a negative environmental impact. They are, hence, increasingly and efficiently replaced with energy harvesting (EH) systems, thus enabling the development of a new class of autonomous wearable devices and resulting sensor networks [3,4]. In fact, EH involves the collection of low-level energy from the environment and its conversion into usable electrical energy.

**Figure 1:**
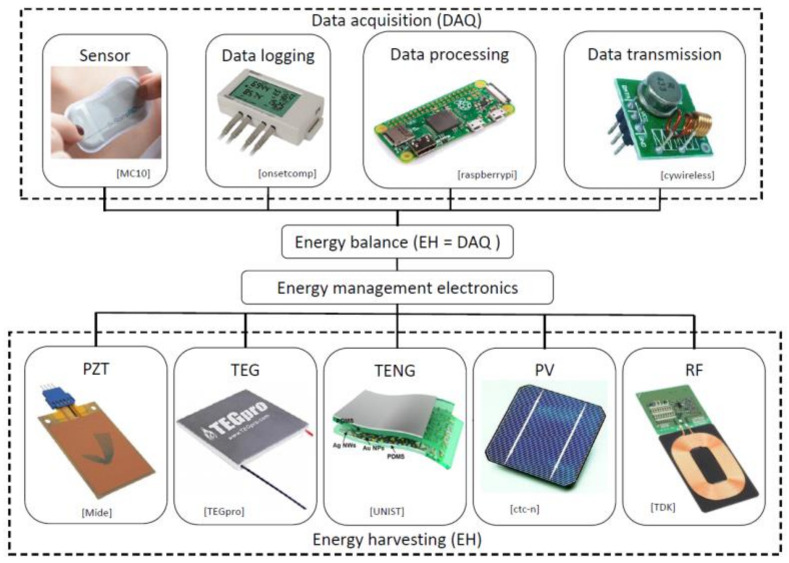
EH integration in autonomous devices with sensors and the respective data processing and communication modules, enabling the creation of sensor networks [8].

The most common ambient energy sources are kinetic—converted via the piezoelectric effect or by using the electromagnetic or triboelectric principles, thermal (waste heat)—collected by using thermoelectric (Seebeck effect) devices, solar or light energy—converted by using photovoltaics, as well as radio frequency (RF)—which can be harvested by using specialized antennas [5,6]. The resulting autonomous EH-powered wearable devices can include various sensors, e.g., heart rate, blood pressure, glucose level, temperature sensors or oximeters, as well as an energy storage element and data processing and communication components, making them suitable for creating autonomous sensor networks aimed at remote patient monitoring and telemedicine, professional athletics, work safety in high risk professions and various IoT or structural health monitoring (SHM) applications [3,4,7,8]. The concept of EH integration in autonomous sensor networks is outlined in Figure 1.

In this framework, kinetic energy, present in all moving systems, also induced by human motion, is a very reliable and copious source of ambient energy. The piezoelectric principle, due to its simplicity, versatility, miniaturization potential and energy density, represents, in turn, a favourable approach to the collection and conversion of ambient kinetic energy [5,6].

**Figure 2:**
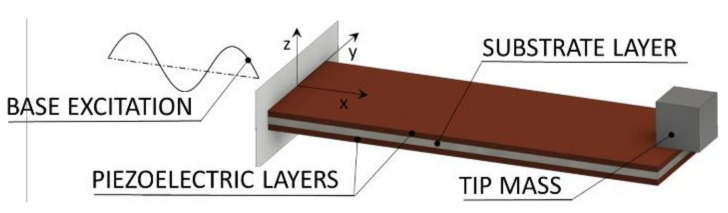
Bimorph piezoelectric energy harvester [3].

Based on a seminal work of Priya in 2005 [9], numerous solutions aimed at human motion EH are recently suggested in literature. The device suggested by Pozzi et al. [10], based on an inverted piezoelectric windmill to utilize the relative movement at the human knee, represents in this frame one of the early solutions. Despite its rather hefty size, the system generated a relatively low power output that, even with some improvements proposed subsequently by the same research group [11], remained limited to the mW range. The introduction of a flywheel, which collects kinetic energy at the human wrist and periodically excites a piezoelectric energy harvester (PEH), resulted then in a new type of rotational EH device [12]. As shown in Figure 2, the most common form of a PEH device used in this framework is a bimorph cantilever of rectangular shape, comprising a metallic substrate with two layers of piezoelectric materials deposited on it. In fact, the bimorph configuration was found to be more efficient compared to the unimorph one [13]. The device is then clamped at the excited end, allowing the bimorph to oscillate—thus deforming the piezoelectric material and generating charge (i.e., voltage). In some cases, a tip mass is attached to the free end of the cantilever in order to increase the displacements and tune the response of the device to the excitation frequency, resulting in higher power outputs [5,6].

In [14] a miniaturized wearable device is then proposed, comprising a flywheel that magnetically excites a single rectangular PEH, whereas in [15] a device, utilizing a flywheel with magnetic plucking, is studied from the standpoint of the relative positions of the excitation magnets. The latter solution is somewhat improved by introducing several thin-film PEHs [16]. Although the performances of the above devices are increased compared to conventional PEHs exposed to human motion excitation, their power output levels are still rather limited, generating merely ~40 μW.

Various innovative approaches have thus been proposed in recent literature. The double pendulum mechanism for leg movement EH, albeit providing a viable approach and showing promising performances (~2 mW), uses a relatively complex motion mechanism [17]. A handheld device, consisting of a rotating flywheel equipped with magnets, interacting with stationary magnets attached to radially placed piezoelectric beams fixed on both ends, results again in a rather bulky and complex device, generating a small amount of power, i.e., 0.18 μW [18].

What is more, a very small number of these studies considers the dynamical stresses present in the piezoelectric materials, and thus the durability of the developed devices [18,19]. This would indicate that a notable number of proposed devices would, in practice, be unable to perform, in the long run, as they were meant to.

Another recent research direction is based on improving PEHs’ performances by changing the geometry of the harvesters. It has thus been shown that the performances of a PEH bimorph can be improved using a trapezoidal shape instead of a rectangular one of comparable width; by inverting the trapezoid, the performances can be enhanced even further [20]. Significant power gains compared to the reference shape, although in the μW power range only, were, in turn, achieved by using complex optimized geometries, with the best performing variation being the one without area constraints [21]. Although the static stress was considered in this case, the overall performances of the PEH are, again, uncertain when the effects of the dynamical stresses in long-term operation, coupled to the narrow cross-section near the fixture of the harvester, are considered. As in the case of the trapezoidal and inverse PEHs, a large portion of the reference rectangular PEH, whose use could potentially lead to further gains in performances, is removed in the optimization process, here, as well.

The process of topology optimization has also been applied to PEHs, both to the thickness of the layers as well as to the surface itself, but the resulting geometries, although providing output voltage improvements, are overly complex, while strength considerations are again generally omitted [22,23].

Miniaturized EH devices are also being studied as means of human motion utilization. Various approaches are being considered in this frame. If compared to a conventional MEMS device, the recently suggested harmonically excited one displays then notable power gains and a reduction of the needed excitation frequency, but its operational frequency of 40 Hz and the low power output (<0.5 μW), limits its potential in wearable applications [24]. A miniaturized rotational harvester using a flywheel and mechanically plucked MEMS cantilevers, enables, in turn, generating 11 μW, decreasing, however, by half with a variation in plucking frequency [25]. Despite representing a promising mean of human motion EH, the low power outputs of such design configurations strongly limit their application field.

Another innovative approach to human motion PEH is the utilization of piezoelectric textiles [26]; while the output powers in this case are again low, the possibility of increasing the amount of worn EH material in the form of clothing could provide means to improve performances. Due to the commonplace treatment of clothing elements (crumpling, washing etc.), the durability of such devices is, however, still an issue that strongly limits their potential applications.

In any case, the proposed solutions clearly show that the collection of the high amounts of kinetic energy due to arm and leg motion is a particularly efficient way of harvesting human motion energy [27]. Numerous potential solutions aimed at using this energy via wearable PEHs are then presented in literature, the most promising ones relying on changing the boundary conditions at the cantilever free end via active tuning or damping control, using the above mentioned approach of optimising the bimorphs’ shape, extending the viable frequency bandwidth by employing several harvesters or complex geometries with bi-stable or nonlinear responses, or, finally, by using a frequency up-conversion (FUC) approach, wherein the cantilever’s free end is plucked and the bimorph is let oscillating at its eigenfrequency [3]. After a careful consideration of these approaches, with particular attention to reliability, technological simplicity and ease of operation, a viable and promising path towards a functional wearable device that utilizes kinetic energy from human motion could be an improved wrist-worn device, using a flywheel to excite a number of optimized PEHs.

This work will, thus, provide an enhanced approach to the employment of such a path, where the whole reference rectangular shape of the PEH is used and segmented into optimized sections. The optimization itself is performed by using design of experiments (DoE) methods. The resulting shapes, combined with a suitable miniaturized plucking mechanism allowing the bimorphs to oscillate at their eigenfrequencies, will provide the basis of an innovative highly efficient wrist-worn EH device aimed at powering autonomous wearable sensors. A detailed study of the strength issues will also be performed to ensure the needed resilience of the proposed class of devices.

An overview of the viability of PEH devices as a power source for wearable applications is therefore given in Section 2 of this work, where the major drawback of this EH principle, as well as a potential solution, are pointed out. Section 3 summarises the influence of the PEH devices’ geometry on their electro-mechanical response, allowing innovative designs to be proposed. The considered PEH shapes are then optimized and their responses are analysed. A detailed description of the experimental setups used to validate the numerical results is given in Section 4. Due to the dynamical nature of PEHs’ operation, stress analyses are performed in Section 5 to determine the maximum excitation levels, while not exceeding the fatigue limit of the piezoelectric material. The results obtained via the described experimental setups are analysed and compared in Section 6. The conclusions of the work are finally drawn in Section 7, where an outlook for future work is provided as well.

## 2. Energy from Human Motion and Frequency Up-Conversion

**Figure 3:**
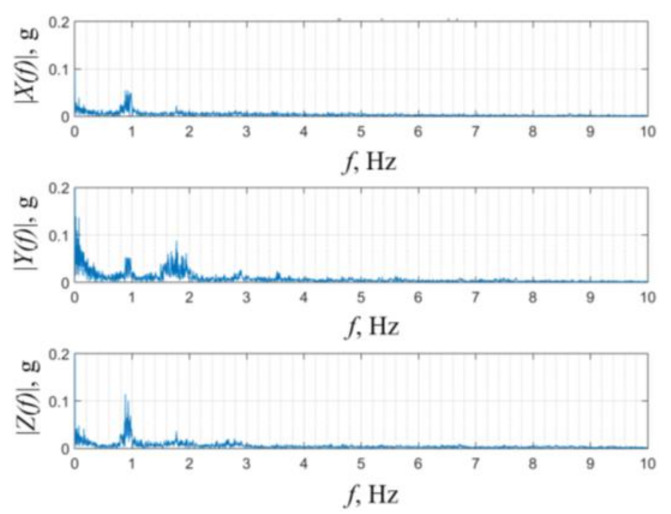
Accelerations during human walking in the frequency domain [30].

Kinetic energy generated by human motion is generally in the form of vibrations or impacts, and, not unlike in several IoT or SHM settings, rather than being periodical, it is characterized by random and chaotic movements. In previous art it was, in fact, established that the conventional activities of daily living (ADL) result in non-periodical excitations of varying amplitudes, with frequencies between ~0.5 and 10 Hz (with a predominant occurrence of lower ones) [28] and with respective maximal acceleration values of up to ~1 g [29]. As shown in Figure 3, for human walking, these ranges of values have been confirmed, also, by studies performed at our premises, in which dominant frequencies of around 1 and 1.8 Hz, with accelerations of ca. 0.1 g, were recorded. In the case of running, values of ~1.3 and ~2.6 Hz, with accelerations of ~0.7 g were, in turn, recorded [30].

On the other hand, it was established that all cantilever-based PEHs are characterized by the same major drawback, i.e., the rapid decrease in conversion efficiency, along with a corresponding decrease of voltage and power outputs, when the excitation frequency moves even slightly away from the eigenfrequency of a specific device [3,5,6]. By introducing PEHs into wearable technologies, this issue, considering the outlined nature of human motion, becomes especially relevant. One of the most promising ways to overcome it is to use the frequency up-conversion (FUC) mechanism [12,31,32], i.e., by converting random human motion into a periodical excitation of the harvesting device by impacting (plucking) the free end of the PEH and letting it oscillate at its eigenfrequency. The FUC approach ensures, thus, that the PEH transduction operates always at the maximum conversion efficiency, despite the random nature of the excitation [12,31,32], significantly facilitating the successful integration of PEH principles into wearables and other applications characterised by random kinetic excitation spectra [3,8,32].

## 3. Geometry Optimization and Influence on the PEH Response

The most commonly used and commercially readily available PEH type is a cantilever with a rectangular planar layout. A considerable amount of studies, investigating the influence of geometry on PEHs’ response is available in literature [3,5,6,12,20,21,22,23], mainly focusing on the increase of PEH power density, particularly important for wearable applications, and the broadening of the excitation bandwidth, resulting in a wider applications domain. In order to expand the thus experimentally validated findings on the possibility of replacing a rectangular PEH with trapezoidal or inverse trapezoidal shapes, resulting in a substantial increase in specific power outputs [20], a numerical model is developed using ANSYS^®^ [3]. The damping coefficients required in the carried finite element (FE) analyses are determined here via uncoupled modal analyses, while, due to the complex interactions induced by the forward and backwards electromechanical coupling effects, the optimal load resistance values are determined via the well-established practice of multiple harmonic analyses [3,33,34,35]. In fact, the approximate equation for determining the optimal resistance [36,37] provides this value for a single set of working conditions, i.e., a single working point.

**Figure 4:**
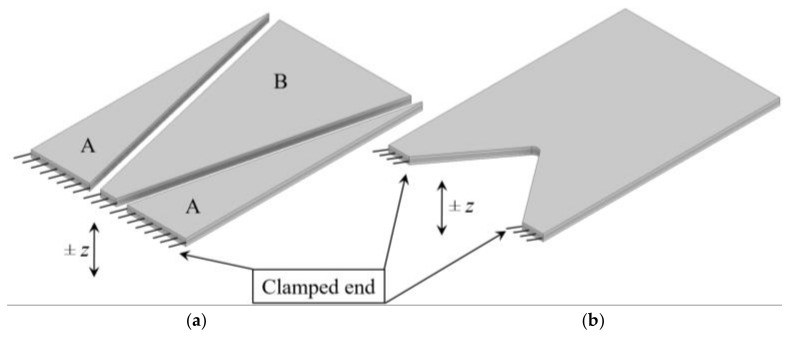
Segmented PEH (**a**) and PEH with a triangular notch at the clamped end (**b**), with the excitation displacement of the clamped end denoted by *z*.

An initial study comprising numerous FE analyses shows hence that, when compared to a conventional rectangular PEH of the same overall surface area, the design configuration segmented in two trapezoidal (A) and an inverted trapezoidal (B) shape (Figure 4a), allows attaining a higher specific power output, since it allows a more efficient utilization of the limited volume available in wearable devices [3,12]. What is more, the maximal power output of each segment can be specifically matched to an electrical load equivalent to that of a diverse wearable sensor [3]. A further performance-enhancing design variant, comprising a triangular notch at the clamped end (Figure 4b), thus increasing the compliance of the harvester and inducing stress concentration effects, is also analysed [12].

The results of the performed study show thus the viability of geometry alteration as means to increase the specific power output of PEHs as well as, especially when coupled to FUC-based loading, to broaden the respective useful excitation spectrum. To determine the ideal PEH dimensions, resulting in the highest power outputs, a more in-depth analysis, comprising a structured optimization process, as described in our previously disclosed studies [12,38] is, however, needed [12]. The bimorph harvesters considered in the optimization process, comprising a stainless steel substrate layer and two PZT-5A piezoelectric layers, are commercially available ones [39]. It has an overall surface area of length (*l*) × width (*w*) = 23 × 15 mm, whereas the thickness of the stainless steel substrate is *t*_s_ = 0.15 mm and of the two PZT-5A piezoelectric layers is *t*_pzt_ = 0.254 mm, making the overall thickness of the bimorph *t*~0.7 mm. The relevant basic electromechanical properties of the used materials are reported in Table 1, while the strength limit of the piezoelectric material will be quantified in Section 5 of this work, where it is used as the relevant criterion for the performed stress analyses. In the performed simulation, a variable resistive load is then connected to the PEHs subjected to a 1 g harmonic excitation [38]. The central composite design of experiments (DoE) algorithm is, hence, employed to generate random combinations of the characteristic dimensions of the segmented and notched harvesters, while taking into account also the technological and practical aspects, i.e., the space needed for clamping and soldering the connections [40]. Via modal and coupled 3D FE harmonic analyses and suitable optimization criteria, the optimal combination of the considered dimensions is therefore determined. The inverted trapezoidal shape has thus an optimal width at its narrow end of 3 mm, and, at its broader end, of 14 mm, with the width of the two trapezoidal half-portions of Figure 4a adjusted accordingly to use the whole available rectangular footprint. The width and height of the notch in the corresponding design configuration are, in turn, respectively 11 and 6.5 mm.

In Table 2, are, then, listed the correspondingly obtained maximal output powers, as well as the respective optimal load resistance values; the comparison of the performances of the optimized PEHs to that of a conventional rectangular PEH, having an overall surface area on which the optimal shapes are based, is also provided. It is, thus, initially established that the maximal output power values (*P*_max_) are obtained using the notched PEH design variant. When the combined power outputs of all the three segments are taken into account, the segmented PEH shows also far better results than the conventional rectangular version. When the specific power output *P*_Smax_, i.e., the maximal power output normalized to the PEH surface area, is considered, the highest values are achieved using the inverted trapezoidal configuration, with the second highest values obtained with the notched PEH. The specific power output of the two trapezoidal segments is lower than that of the rectangular shape, but it should be viewed as a further addition to the already high specific power attained via the inverted trapezoidal shape [38].

When the free end of each studied PEH device is subject to an identical plucking deflection *δ*_z_ and their resulting free oscillations at the respective eigenfrequencies are preliminarily calculated via FE coupled transient analyses, it is, in turn, concluded that the maximum peak-to-peak voltage generated by the inverted trapezoidal segment alone at its optimal load resistance *R*_L opt_ is comparable to that generated by the conventional rectangular PEH device. When the surplus voltage generated by the two trapezoidal segments is also considered, the segmented PEH clearly outperforms the conventional one. The maximum peak-to-peak voltage values generated by the notched PEH is comparable to that of the inverted trapezoidal shape. This data supports, therefore, fully the practicability of the integration of the optimized PEH shapes with the FUC excitation approach [38], which will be investigated in more detail in the below treatise.

**Figure 5:**
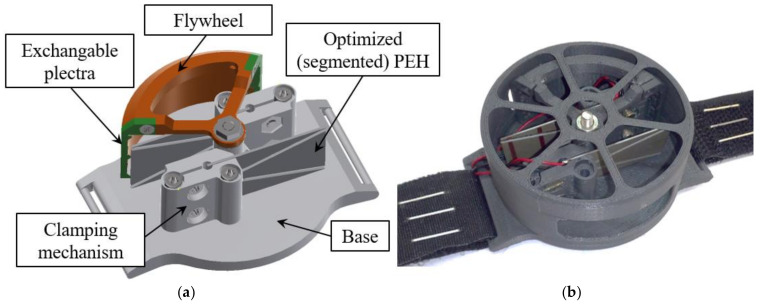
Schema of the devised watch-like wearable PEH device with two optimized segmented bimorph cantilevers (**a**) and the first prototype of the device (**b**).

The illustrated approach is then at the basis of a prototype *ϕ* = 50 mm watch-like wearable device shown in Figure 5, that the Precision Engineering Laboratory of the Faculty of Engineering of the University of Rijeka, Croatia [41] is developing in collaboration with medical institutions, where FUC is achieved via a rotating flywheel equipped with multiple plectra. The prototype being developed includes optimized segmented bimorph harvesters, as determined in the above analyses.

## 4. Experimental Setups

The performed extensive static, modal, harmonic, and transient numerical FE analyses have to be validated by suitable experimental measurements. Two different measurement setups are thus developed at the Precision Engineering Laboratory [41], i.e., a harmonic setup and the FUC setup. The performed experiments will then provide means to fully characterised the optimized PEHs to be used in the suggested wearable prototype.

**Figure 6:**
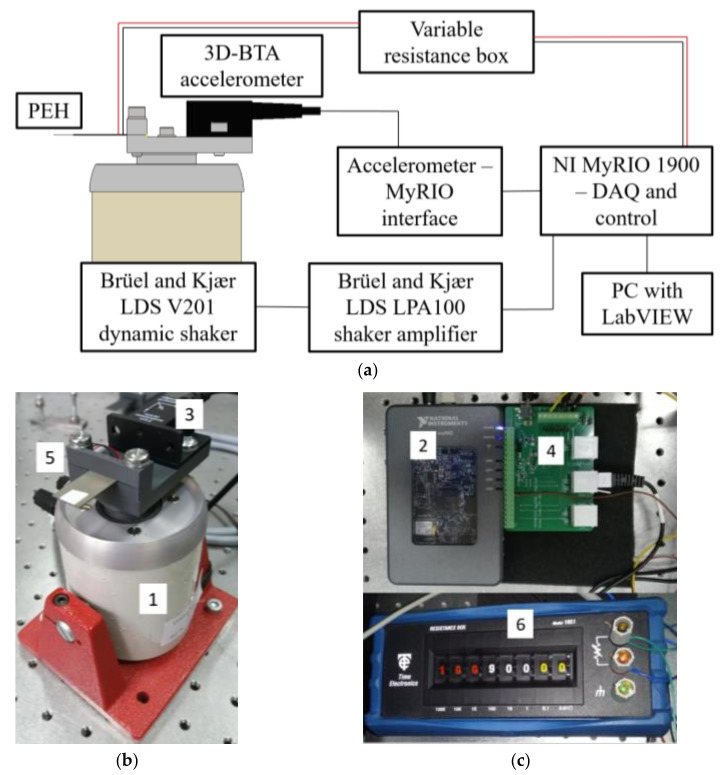
Schema of the experimental setup for harmonic analyses (**a**) with the actual execution showing the Brüel and Kjær^®^ shaker with the accelerometer and the bimorph harvester [38] (**b**), as well as the control, DAQ and variable resistor units [38] (**c**).

The first setup, schematically shown in Figure 6a, is employed to induce a harmonic excitation to the clamped PEH bimorph over a predefined frequency range. It is based on the Brüel and Kjær^®^ LDS V201 electrodynamic permanent magnet shaker (indicated in the Figure 6b with 1) coupled with the LDS LPA100 power amplifier. The excitation parameters are controlled via an NI LabVIEW^®^ virtual instrument operating on the NI MyRIO 1900 device (2), which is used for data acquisition (DAQ) as well. The acceleration of the shaker and of the fixture of the PEHs, is measured via the Vernier^®^ 3D-BTA accelerometer (3) connected to the Vernier^®^ BT-MDAQ adapter (4). The tested *l* × *w* × *t* = 23 × 15 × 0.7 mm PEH (5), is finally connected to the DAQ unit via the variable resistance box (6) [38].

**Figure 7:**
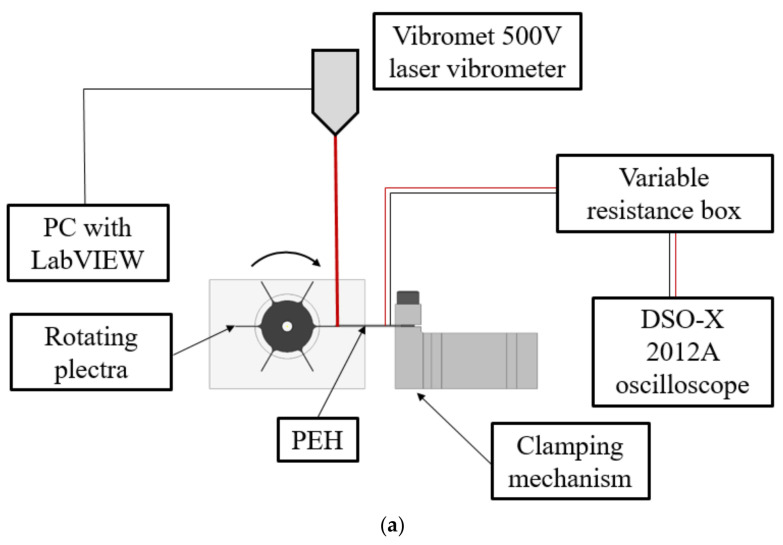

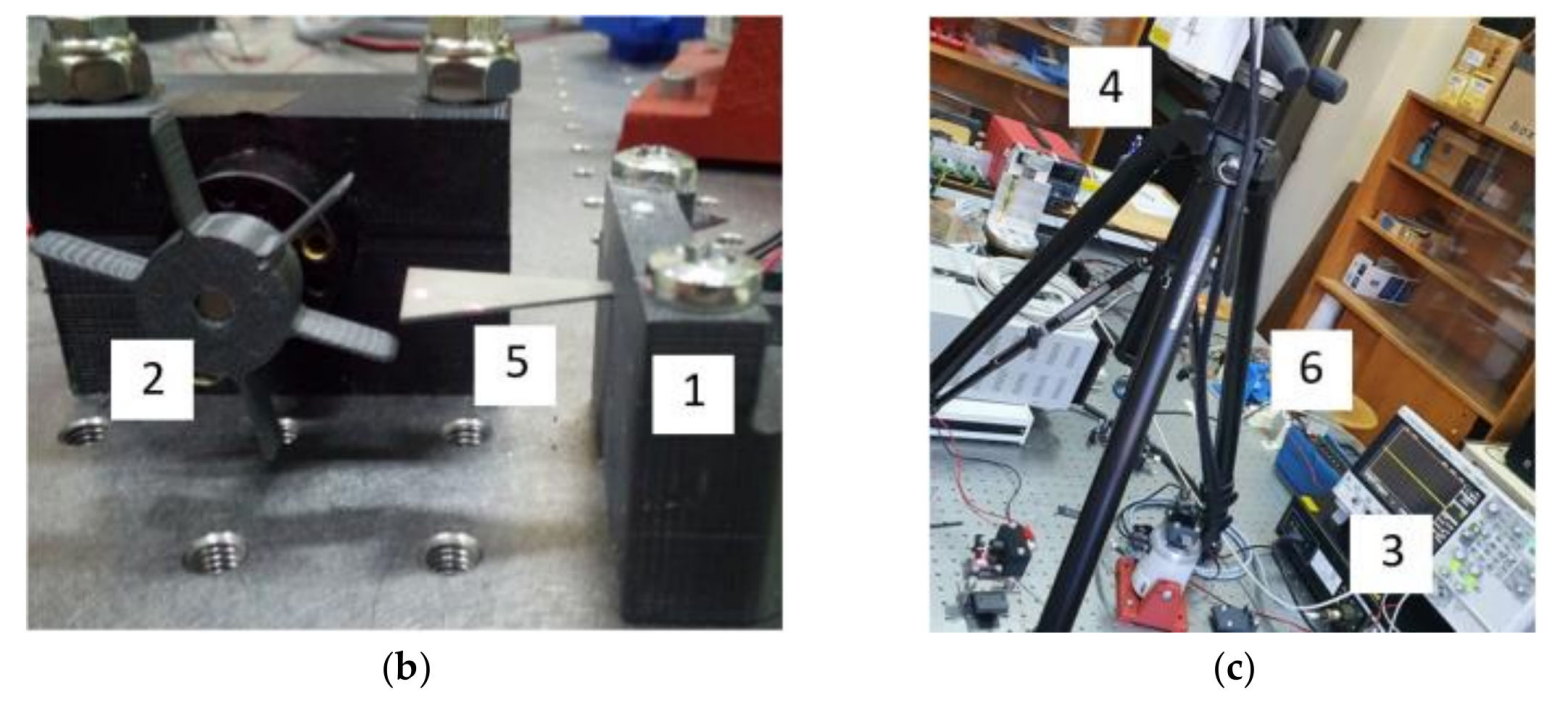
Schema of the FUC experimental setup (**a**) with the actual execution showing the plucking mechanism [38] (**b**) and the DAQ system [38] (**c**).

The setup aimed at emulating the FUC excitation approach, shown schematically in Figure 7a, comprises, in turn, the 3D-printed PEH clamping mechanism (indicated in Figure 7b with 1) and the 3D-printed rotating exchangeable plectra mounted on the shaft of a DC motor (2). A purely mechanical (i.e., not magnetic) plucking mechanism is used, here, to prevent the possible damping effects of magnets on the oscillating PEHs [15,38]. The voltage output generated by the tested PEH (5) is measured by employing the Agilent^®^ DSO-X 2012A oscilloscope (3), while the displacement of the PEH’s free end is acquired by using a Metrolaser^®^ Vibromet 500V laser doppler vibrometer (4). The PEH itself, having the same overall dimensions as in the case of the harmonic analysis setup, is connected to the oscilloscope via the variable resistance box (6) [38].

## 5. Strength Analysis of Optimized PEH Shapes

During regular operation, a kinetic PEH is subjected to dynamical working conditions. These conditions induce dynamical stresses of the used brittle piezoelectric material. Special attention is thus to be devoted to the fatigue lifetime of the considered PEHs, which is almost completely neglected in prior art. In fact, the criteria of maximum power or voltage output alone, most commonly used in literature [21,28,32], can be relevant only when a small number of operational cycles suffices for a particular application. When, however, a bimorph PEH device is intended for wearable or other devices with common dynamical excitation conditions, the maximum power or voltage outputs can be viable criteria only when the stress levels are lower that the fatigue limit of the used piezoelectric material, i.e., for PZT-5A, *R*_d_ = 55 MPa [42]. FE stress analyses are thus performed in ANSYS^®^ for the conventional rectangular PEH shape, used as a reference, and for the considered optimized PEH shapes.

**Figure 8:**
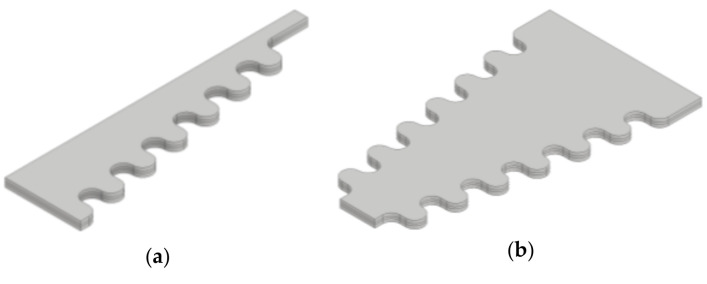
Trapezoidal (**a**) and inverted trapezoidal (**b**) PEH segments with wavy edges (added stress concentrators).

**Figure 9:**
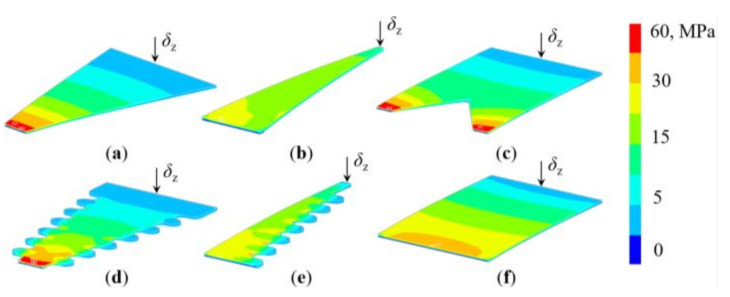
FE calculated stresses in the piezoelectric layers of the PEHs for a *δ*_z_ = 0.5 mm free-end deflection: inverted (**a**), trapezoidal (**b**), notched (**c**), wavy inverted (**d**), wavy trapezoidal (**e**) and conventional rectangular shape (**f**).

In this frame, a variant of the segment harvester shape, aimed at further increasing the power output by introducing wavy contours (notches) along the edges of the segments, as shown in Figure 8, is considered, as well. The assumption is that in this configuration the bigger stress in the piezoelectric material will result in a boost in charge generation, and thus in an increase in voltage and power outputs. In all the performed FE analyses one end of the PEH is then fixed, i.e., clamped, while the free end, without tip mass, is statically bent, with deflections *δ*_z_ ranging from 0.05 to 1 mm, allowing the resulting stresses to be obtained (Figure 9). As expected, the highest stresses occur at the fixture, with a more uniform stress distribution along the trapezoidal shapes (Figure 9b, e). The effect of the wavy edges, i.e., of the stress concentrators on the segmented shape, can, in turn, be clearly observed in the areas of increased stress levels, particularly in Figure 9e. The stresses are redistributed, in this case, towards the concentrators and away from the fixture, making the overall stress distribution more uniform. These considerations will have a significant impact on the power output of the optimized PEHs, as elaborated in Section 6 below.

**Figure 10:**
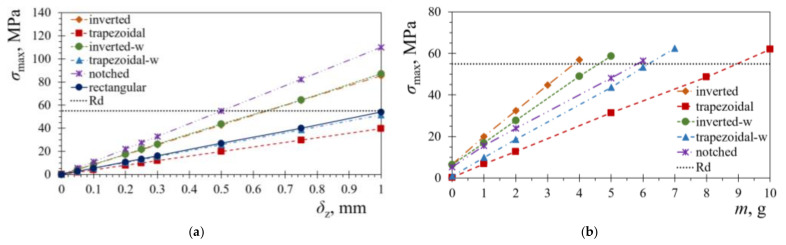
Piezoelectric material bending stresses of the considered PEH shapes: for various tip displacements *δ*_z_ (**a**) and for various tip masses *m* (**b**) when the PEHs are subject to harmonic excitations.

The thus attained maximum bending stress values *σ*_max_ in the piezoelectric layers, as determined via the performed FE analyses, are reported in Figure 10a versus the respective tip displacements *δ*_z_. To determine the maximum allowable deflection of the considered PEH shapes, the fatigue bending limit of the used PZT-5A piezoelectric material *R*_d_ is also marked on the graph. From the stress data analysis, it can be concluded that, in order to keep the stress levels below the dynamical strength limit, the tip deflection *δ*_z_ of around 0.5 mm can be applied to the notched shape, while *δ*_z_ = 0.6 mm is applicable to the inverted trapezoidal shape with and without stress concentrators. The trapezoidal shape with stress concentrators and the conventional rectangular shape can be subjected to *δ*_z_ = 1 mm, while an even larger deflection can be applied to the trapezoidal shape with a straight edge. By limiting the plucking deflection of the optimized PEHs to these values, the fatigue safety of the piezoelectric layers can be assured, and thus long-lasting operation of the device can be achieved.

Further numerical analyses can be performed in ANSYS^®^ when the tip masses *m* are introduced as well, while each of the considered PEH shapes is subjected to a 1 g harmonic excitation. The optimal tip-mass values, corresponding to the fatigue-strength limit, can hence be determined. The thus obtained results are shown in Figure 10b, where it can be observed that, as could have been expected, the largest tip mass of *m* = 9 g can be safely attached to the trapezoidal PEH, while only *m* = 4 g can be used with the inverted trapezoidal shape. The rectangular shape, not shown in Figure 10b for reasons of clarity, can securely withstand *m* = 25 g that cannot, however, be packed in a volume suitable for a practical use in wearable (wrist-worn) applications, even when high-density materials, such as tungsten, would be used to obtain it.

**Figure 11:**
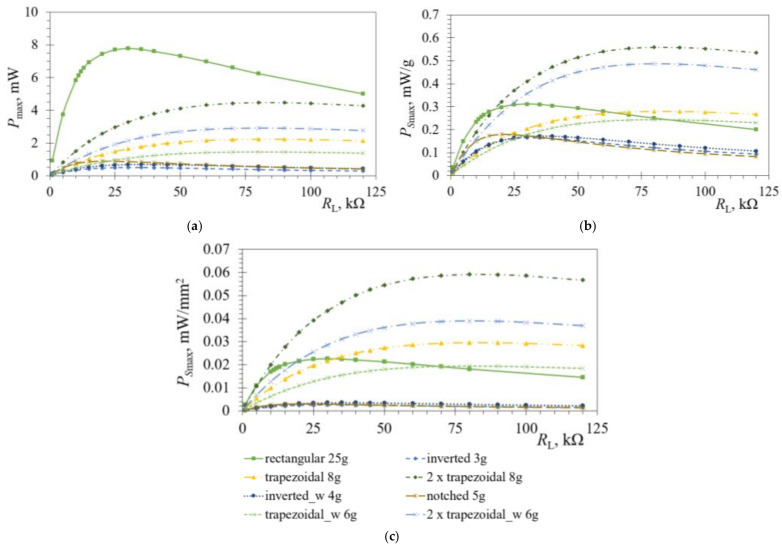
Coupled harmonic responses of the various PEH shapes with optimal tip masses *m*: *P*_max_ (**a**), *P*_Smax_ normalised by *m* (**b**), and *P*_Smax_ normalised by the PEH surface area (**c**) vs. *R*_L_.

Since the behaviour of the bimorph PEHs changes significantly with every variation of the tip mass value, coupled harmonic analyses are performed next for the considered geometries with an optimal tip mass attached to the free end. A sweep through load-resistance values *R*_L_ is then conducted, again, to determine the optimal resistances and the respective maximal power outputs *P*_max_. The thus obtained results are shown in Figure 11a. Figure 11b,c show, in turn, the specific power values *P*_Smax_ normalized, respectively, by the tip-mass and the PEH surface-area values.

In Figure 11 it can, hence, be observed that, when only the maximal power output levels are considered, the best performances are attained with the rectangular PEH loaded with *m* = 25 g. As already pointed out, if such a device would be employed in wearables, the size of the harvester would be unsuitable due to the large volume of the tip mass. When the power outputs are normalized by the tip masses (Figure 11b), the sum of the powers of the two trapezoidal segments is the highest, with the output of a single trapezoidal segment being comparable with that of the rectangular PEH. A similar behaviour can be observed if the maximal output powers are normalized by the surface area of the PEHs (Figure 11c). It can also be noted, here, that in all the cases of Figure 11 the performances are reduced for most of the optimized shapes, i.e., the inverted trapezoid, the notched shape as well as the shapes with stress concentrators along the edges. This is due to the limited ability of these shapes to withstand larger tip masses, while ensuring a continuous operation in dynamical working conditions. If, however, the specific requirements of PEH devices for wearable applications, in terms of high specific power outputs and low masses and volumes, as well as limited tip deflections are taken into account, the combination of two trapezoidal and one inverted segment represents surely a very practicable choice. What is more, the absolute power levels generated at the optimal loads by the developed optimised PEHs with tip masses (Figure 11a), i.e., 0.5 mW for the inverted trapezoidal shape, 0.9 mW for the notched one and 2.2 mW for a single trapezoidal shape, taking into account the respective duty cycles, are more than enough to power a combination of wearable sensors, data-processing and communication components, as established in [3], thus, enabling fully operational and long-lasting autonomous wearable device.

## 6. Results and Discussion

The described experimental setups were used to measure the performances of the rectangular and of the optimized PEHs undergoing both harmonic and plucking excitations. The thus obtained responses are then graphically depicted and compared to the numerical results.

### 6.1. Damping Ratio

**Figure 12:**
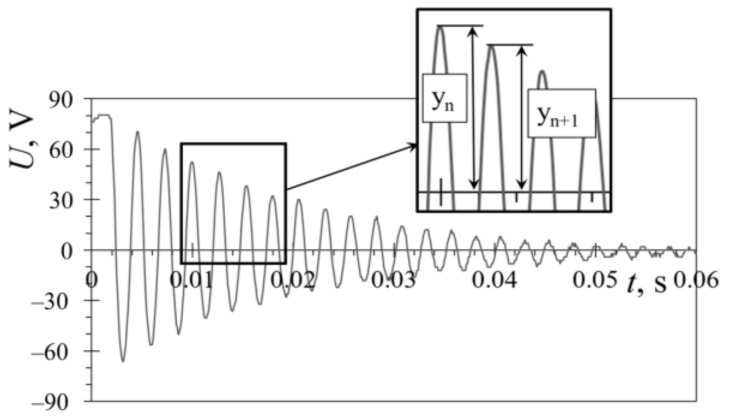
Typical mechanical response obtained by plucking the PEH’s free end.

The first step in validating the numerical models is determining the damping ratio of the harvesters, by measuring the pure mechanical response of the bimorph PEHs. Two excitation approaches were, therefore, used to acquire the data. Firstly, the harvester is excited by plucking its free end, and letting it oscillate at its eigenfrequency. The second approach, used to have a better control of the excitation process, is to excite the PEH fixture by using the electrodynamic shaker operating at the eigenfrequency of a specific PEH, stopping the shaker, and letting the harvester oscillate freely. In both cases, the displacement amplitude of the PEHs’ free end is measured via a Metrolaser^®^ vibrometer. A typical mechanical response attained in one of the measurements is shown in Figure 12. Based on the thus acquired data, the amplitudes of two consecutive response peaks *y*_n_ and *y*_n+1_ are quantified, allowing the logarithmic decrement *δ* to be calculated as:

(1) $$\delta =\ln\left(\frac{y_{n}}{y_{n+1}}\right)$$

The damping ratio *ζ* is, in turn, determined as:

(2) $$\zeta =\frac{\delta }{\sqrt{4\cdot \pi^{2}+\delta^{2}}}$$

When a commercially available rectangular PEH [39] was considered, a slight difference of 3% was found when comparing the damping ratios obtained via the two approaches, i.e., *ζ* = 0.03 in the case of plucking, and *ζ* = 0.031 in the case of shaker excitation. The thus determined damping ratios were then used to calculate the Rayleigh damping coefficients, essential for the harmonic and transient FE analyses [43].

### 6.2. Model Validation

If the numerical models are to be considered as a suitable tool for the development of innovative PEH design configurations, they need to be validated first. Before the validation itself, detailed measurements of the PEH layer thicknesses were made. The validation itself comprised, then, an FE mesh sensitivity analysis, followed by the validation of the harmonic and transient results.

#### 6.2.1. Bimorph PEH Layer Thickness Measurement

**Figure 13:**
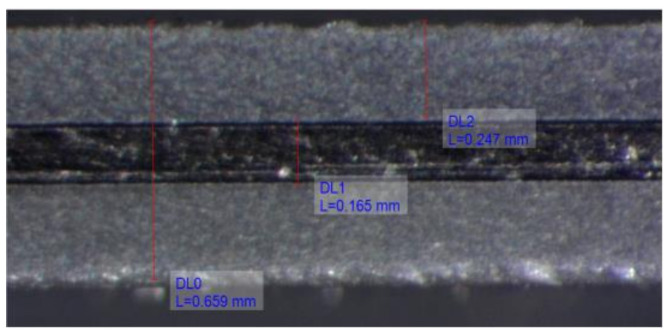
An example of the layer thickness measurement.

To be able to accurately model the bimorph PEH devices, the thicknesses of the respective constituent layers have to be precisely measured. Measurements were hence carried on at the Precision Engineering Laboratory [41] by employing the Olympus^®^ SZX16 optical stereomicroscope equipped with a digital camera. A set of images, such as the one shown in Figure 13, was thus captured under 180× magnification, and the individual layers of the commercial harvester [39] were measured using the calibrated image-analysis software. Following a simple statistical analysis, it was, hence, concluded that the bimorph PEH could be modelled with the metallic substrate layer thickness *t*_s_ = 0.163 mm (with the respective deviation *σ* = ± 0.0043 mm) and the piezoelectric layer thicknesses *t*_PZT_ = 0.251 mm (*σ* = ± 0.0045 mm). A slight difference, with respect to the nominal thickness values as reported in Section 3, was, therefore, observed, which, in turn, due to exponential correlation between the thickness of the harvester and the respective area moment of inertia of its cross-section, had a significant impact on the dynamical response of the device [5,6].

#### 6.2.2. Mesh Sensitivity Analysis

Since the accuracy of the FE model can be significantly influenced by the type of elements used in modelling, as well as the element size (mesh density) [44], a sensitivity analysis was, therefore, performed for two different element types (i.e., hexahedral (brick or cube) and tetrahedral elements) of varying densities. The latter was implemented by varying the edge-element length, set between 0.25 and 2 mm. What is more, in the case of hexahedral elements, two different methods of volume bonding (using the ANSYS^®^ “glue volumes” function and the merging of neighbouring nodes between the substrate and piezoelectric layers) were studied. The relative errors of the various models were then assessed by comparing the solutions with different meshes, in terms of the values of the first eigenfrequency *f*_1_ obtained via the FE models for the rectangular PEH, with the experimental values of *f*_1base_ = 539.38 Hz (*σ* = ±1.47 Hz), obtained in the case of base excitation, and *f*_1FreeEnd_ = 535.15 Hz (*σ* = ±1.04 Hz), attained for the free-end excitation. The slight 0.79% difference of these two approaches is mainly due to PEHs’ clamping implementation, affecting the results. The relative errors *e*_r_ of the modal numerical results with respect to experimental data for both excitation types, are then shown in the following Tables. The mesh-sensitivity validation, using the merged nodes and the hexahedral elements, is reported in Table 3, while the same, using glued volumes with hexahedral and tetrahedral elements, is given in Table 4 and Table 5.

Based on the reported data, it can be concluded that the most accurate results were achieved by using elements with an edge length of around 0.5 to 0.75 mm. It is also important to note that the smaller element sizes, i.e., those <0.5 mm, resulted in significantly extended computational times. The hexahedral elements then better matched the experimental data (*e*_r_FreeEnd_ = 0.06% for the 0.75 mm edge length) as compared with the tetrahedral ones of the same size (*e*_r_FreeEnd_ = 1.58%). On the other hand, the layer-bonding method did not seem to significantly affect the results for element sizes < 1.75 mm.

Given these considerations, hexahedral elements with a 0.75 mm edge length were used in the subsequent harmonic and transient analyses where possible, while tetrahedral elements of a comparable size were used when a specific geometry necessitates it. The layers were, in turn, bonded using the “glue volumes” function, since it is a much quicker and simpler bonding method that does not significantly affect the results.

#### 6.2.3. Harmonic Response Validation

**Figure 14:**
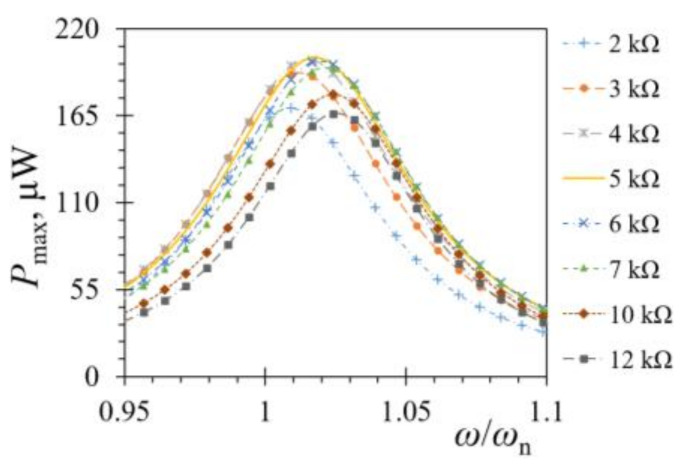
FE obtained powers at various load resistance values.

Numerical FE harmonic analyses were conducted next, by sweeping through an excitation spectrum around the previously determined first eigenfrequency of the rectangular bimorph PEH. The model boundary conditions (excitation, clamping distance and parallel electrical coupling) were set as close as possible to the experimental ones, with the electrical connections and electrodes being considered ideal, i.e., having zero resistance. To determine the maximal power outputs, and thus assess the optimal load resistance, the harmonic analyses were then conducted with varying load resistances. As it can be observed in Figure 14, *R*_L opt_ = 5 kΩ was thus determined for the rectangular PEH.

**Figure 15:**
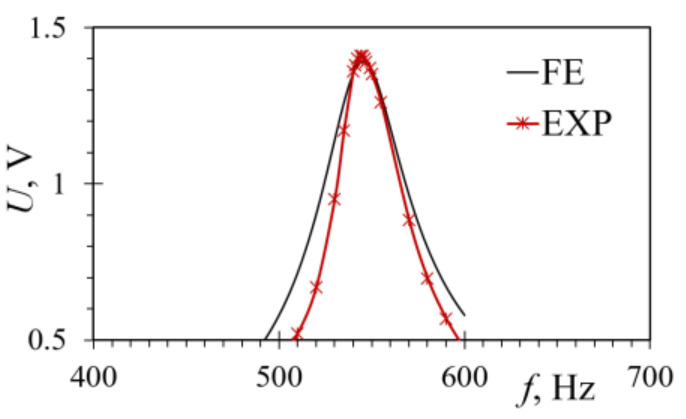
Comparison of experimental and FE voltage output values of a harmonically excited rectangular PEH device.

The experimental data, acquired by using the measurement setup described in Section 4, is, in turn, shown in Figure 15, where it is compared to the FE numerical results, displaying a close match. The maximum voltage output of 1.41 V was achieved experimentally at 544 Hz, while the highest voltage at the same frequency obtained via the harmonic FE model was 1.39 V, resulting in a difference of merely ~1.4%. Despite the good match in the obtained eigenfrequencies and the maximal voltages, there are, however, some discrepancies in the overall shape of the response curves, particularly away from the coupled electromechanical eigenfrequency. This could be attributed to slight experimental excitation control errors as well as clamping inaccuracies (a small difference in cantilevers’ length can have a noteworthy influence on PEHs’ responses). The evaluation of the damping ratio, and the subsequent calculation of the Rayleigh damping coefficients, as well as some limitations in the ANSYS^®^ modelling of the coupled electromechanical piezoelectric effects evidenced in [45], could each, in turn, have had an effect on the numerical results.

**Figure 16:**
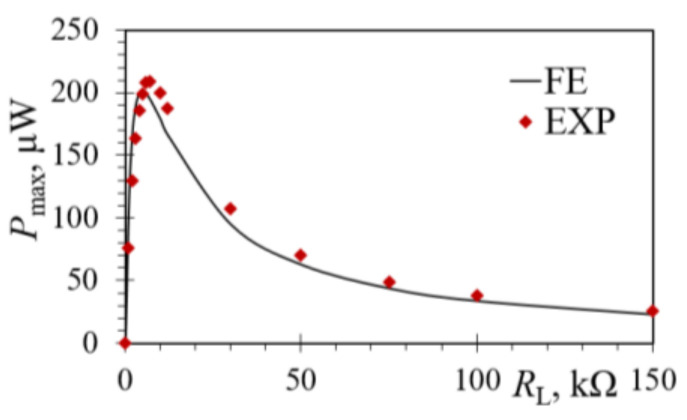
Comparison of experimental and FE power outputs vs. *R*_L_ for a harmonically excited rectangular bimorph PEH device.

In addition to the experimental measurements at the optimal load resistance value of 5 kΩ, the power outputs were measured and determined numerically too, as shown in Figure 16, for load resistances varying in the range from 1 up to 150 kΩ. The thus obtained results show a close match, with a ~3.5% difference in the maximum power value and a shift of the peak determined experimentally towards *R*_L_ = 6 kΩ. These small differences can possibly be attributed to the additional resistances and other minor inaccuracies present in the non-ideal experimental system, neglected in the FE model, due e.g., to the clamping system, excitation control, electrical connections and similar effects.

#### 6.2.4. Transient FUC Response Validation

**Figure 17:**
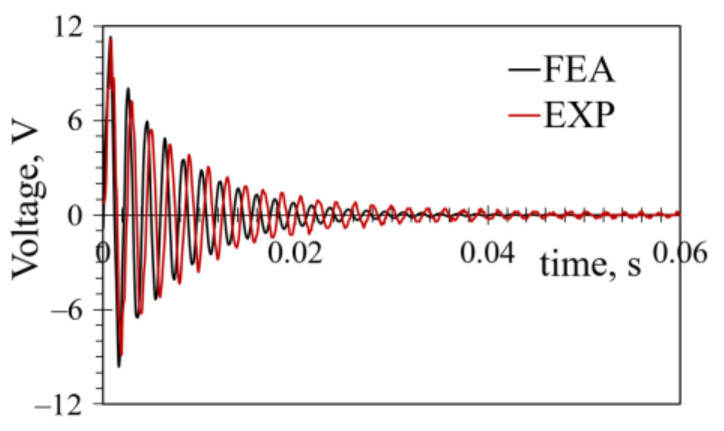
Comparison of experimental and transient FE FUC responses for a rectangular bimorph PEH excited by plucking its free end.

To validate the FE model from the FUC perspective, transient analyses of a rectangular bimorph PEH, excited by plucking its free end, were performed next. The boundary conditions remained the same as in the harmonic model, while the excitation was introduced as a displacement *δ*_z_ of the free end of the harvester. To exclude the possibility of overstressing the PEHs during the extensive experimental tuning of the FUC setup, *δ*_z_ = 0.6 mm was, initially, chosen. The numerical and experimental tests were then carried on at the determined optimal load resistance *R*_L opt_ = 5 kΩ. The obtained results are shown in Figure 17. A close match of the experimental and the FE responses can thus be seen, particularly in the first five cycles, where the highest voltages, i.e., the majority of the power, is generated. The maximum measured peak-to-peak voltage was *U*_p-p_EXP_ = 20.1 V, whereas the corresponding FE value was *U*_p-p_FE_ = 21.26 V, giving a difference of ~5%. The differences between the FE and experimental results in the next four cycles ranged from ~1% to ~9%. A better matching in the subsequent cycles could be achieved by tuning the damping ratio and the respective damping coefficients, but this would cause a significant mismatch in the foremost section of the coupled electromechanical response.

**Figure 18:**
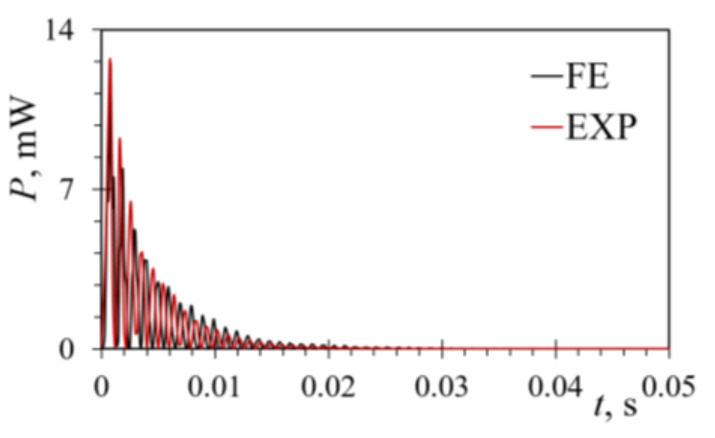
Experimental and transient FE FUC power outputs for a rectangular bimorph PEH excited by plucking its free end.

The power generated via the FUC-based excitation at *R*_L opt_ = 5 kΩ was calculated from the thus-obtained voltages and is displayed in Figure 18. The hence obtained average power values over a 0.05 s time interval were *P*_av_FE_ = 0.454 mW and *P*_av_EXP_ = 0.468 mW, with a slight difference of ~3%. For the deflection of *δ*_z_ = 0.6 mm, the attained maximal powers were, in turn, *P*_max_FE_ = 12.53 mW and *P*_max_EXP_ = 12.42 mW, with a difference of merely 0.88%.

Based on these results, it can be concluded that the used FE models match well the experimental responses in the cases of both excitation approaches, and they can, therefore, be unhesitatingly employed to develop optimized PEH designs.

### 6.3. Optimized PEH Responses

Based on the described optimization process, five different PEH bimorph device designs with optimized shapes were made by cutting commercially available rectangular PEHs [39] with water-jet technology [46]. The responses of the harvesters were then assessed by plucking their free ends, using the FUC experimental setup. The output voltages were measured at the optimal load resistances of each individual device, and the resulting power outputs calculated.

The first eigenfrequencies *f*_1_ of all the optimized PEHs were assessed first, and compared with the respective FE modal analysis results (Table 6). The measured eigenfrequency values (depending on the shape, being between ~325 and ~930 Hz) match closely the FE results. In fact, in most cases the difference is <1%. Only in case of the trapezoidal PEH with wavy edges, there is a slightly increase in this difference, which is still <5%; this could be attributed to clamping inaccuracies induced by the small size and the geometrical complexity of this particular harvester. 

**Figure 19:**
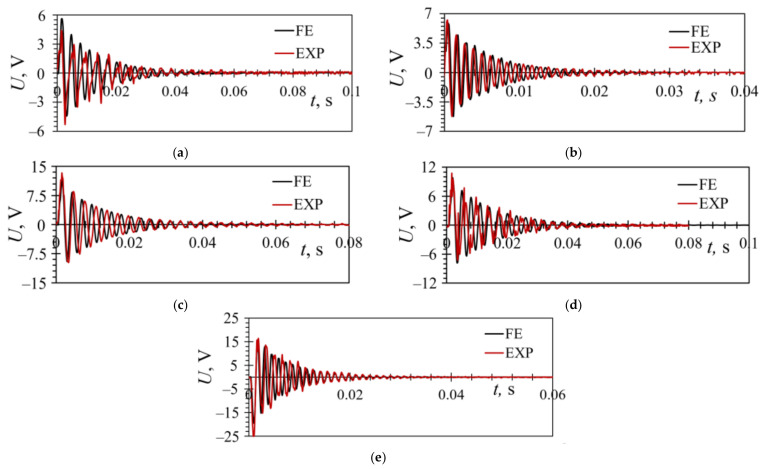
Comparison of experimental and transient FE responses of the PEHs excited by plucking for: an inverted trapezoidal (**a**), a trapezoidal (**b**), a notched (**c**), an inverted wavy trapezoidal (**d**) and a trapezoidal wavy (**e**) bimorph PEH.

The FUC responses for each individual PEH were experimentally assessed next, and the resulting voltages compared to the transient FE results. The initial displacement of the free end of the harvesters, obtained in all the cases by plucking the harvesters with the same rectangular 3D-printed plectrum, while the DC actuator rotated at the same speed, was measured during each experiment, and the obtained results for the analysed PEHs, considerably improved in accuracy and consistency with respect to the preliminary ones reported in [38], are depicted in Figure 19, exhibiting, in all cases, a close match. The initial free end displacement values were, again, limited due to strength considerations (cf. Section 5).

In Figure 19a are shown the responses for the inverted trapezoidal bimorph harvester. The displacement of the free end was *δ*_z_ = 0.27 mm, while the optimal load resistance was *R*_L opt_ = 13 kΩ. The largest measured peak-to-peak voltage was *U*_p-p_EXP_ = 9.66 V, while the corresponding FE value was *U*_p-p_FE_ = 10.04 V (the difference is ~3.8%). The oscillation period of the experimental and FE results was similar: *t*~0.06 s.

The responses for the trapezoidal PEH is, in turn, displayed in Figure 19b. In this case *δ*_z_ = 0.2 mm, *R*_L opt_ = 7 kΩ, *U*_p-p_EXP_ = 11.46 V, *U*_p-p_FE_ = 11.07 V (difference of ~3.5%), and the oscillation period was *t*~0.03 s.

The response for the PEH device with a triangular notch at the clamped end is displayed in Figure 19c. The characteristic performances were: *δ*_z_ = 0.47 mm, *R*_L opt_ = 7 kΩ, *U*_p-p_EXP_ = 23.1 V, *U*_p-p_FE_ = 21.4 V (difference of ~7.5%), and *t*~0.08 s.

The responses for the inverted trapezoidal harvester with stress concentrators along the edge of the bimorph and the corresponding trapezoidal PEH cantilever are, finally, given in Figure 19d, e, whose respective characteristic values were: *δ*_z_ = 0.48 mm, *R*_L opt_ = 12 kΩ, *U*_p-p_EXP_ = 17.9 V, *U*_p-p_FE_ = 17.61 V (~1.6% difference), and *t*~0.06 s (Figure 19d), and *δ*_z_ = 0.38 mm, *R*_L opt_ = 13 kΩ, *U*_p-p_EXP_ = 41.62 V, *U*_p-p_FE_ = 40.98 V (1.5% difference), *t*~0.03 s (Figure 19e).

The thus obtained maximal peak-to-peak voltages *U*_max_p-p_, as well as the average, the maximal and the maximal specific powers *P*_av_, *P*_max_, and *P*_s_max_, are listed in Table 7. The average powers were calculated, here, over the reported oscillation periods for the respective bimorph PEHs, while all the reported data were obtained for the stated initial deflections *δ*_z_, as obtained experimentally in the described conditions.

### 6.4. Discussion

**Figure 20:**
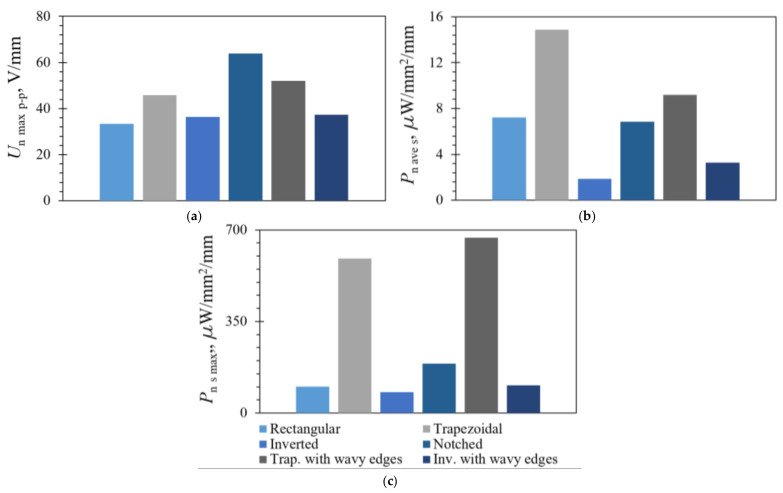
Normalized maximal voltages (**a**), as well as respectively calculated specific average (**b**) and specific maximal (**c**) power outputs for the considered bimorph PEH shapes.

As stated, in the employed experimental setup the displacements of the free end were attained by plucking all the used bimorph harvesters with the same rectangular 3D-printed plectrum rotating at the same speed. This resulted in a different *δ*_z_ for each PEH shape, thus making comprehensive comparison difficult. Shown in Table 8, the obtained voltages and powers were, hence, normalized by *δ*_z_, resulting in a dimensionality (*U*_n_max_p-p_, V/mm; *P*_n_xx_, mW/mm) that, as evidenced also in the respective graphical representation of Figure 20, provided a better means of comparing responses between the used piezoelectric harvesting devices.

It can thus be observed that, in terms of the normalised output voltages, all optimized bimorph PEH devices, if compared with the conventional rectangular shape, generated larger outputs, the highest value of which was obtained from the notched PEH. An increase in output voltages can also be noted in the PEHs with wavy edges, compared with their counterparts, without added stress concentrators (Figure 20a).

When the normalised average power is considered, as shown in Table 8, the highest output was obtained from the rectangular PEH, while the optimized bimorph PEHs generated less power. When, however, the average power is normalized over the respective PEH surface area (Figure 20b), the specific normalized average power output of both trapezoidal PEHs is noticeably higher than that of the rectangular PEH, while the values for the notched PEH are slightly lower but comparable (~5%) to those of the rectangular one. The specific normalized average power of both inverted trapezoidal PEH shapes was lower than that of the rectangular one but, as already pointed out, this output is, in practice, an addition to that of the respective trapezoidal segments.

When, finally, the maximum power outputs, calculated from the normalized voltages and divided over the surface area of the respective PEHs, were observed, as shown in Figure 20c, it could be noted that, especially, both the trapezoidal PEHs, but also (less pronouncedly) the notched PEH, significantly outperformed the conventional rectangular bimorph. Both optimized inverted segments, by themselves, generated a specific normalised maximum power output comparable to that of the rectangular shape, with the inverted PEH with wavy edges having even slightly outperformed it. Moreover, as with the maximal voltages, a noticeable increase in the normalised specific maximal powers was obtained for both PEHs with added stress concentrators, compared with those without them.

If the determined optimal bimorphs’ design configurations would then be excited by the respective maximal allowable initial free end displacement *δ*_z_max_, as determined in Figure 10a in Section 5, the corresponding maximal power outputs and the average power outputs during the matching oscillation periods would be those reported in Table 9.

When the thus-obtained power outputs of the segments with straight edges, excited by the respective maximum allowable displacements, were summed, and the resulting performances observed for the segmented device as a whole, the maximum and average power output values were, respectively, *P*_max_*δ*zmax_ = 134.06 mW and *P*_ave_*δ*zmax_ = 3.38 mW, which, when compared with the performance of the rectangular PEH reported in Table 9, represents a significant improvement. Due to the increased stress levels and, therefore, the resulting limited allowable initial free-end displacements, the summed performances of the segments with stress concentrators, in terms of average power output, were slightly lower than those of the rectangular bimorph, i.e., *P*_ave_*δ*zmax_ = 1.76 mW, but, even in this case, the maximal power output exceeded that of the rectangular PEH, since it summed to *P*_max_*δ*zmax_ = 112.1 mW.

## 7. Conclusions and Outlook

The possibility of using energy harvesting, especially the respective piezoelectric approach, in wearable sensor networks, was analysed in this work, along with a detailed review of the state-of-the-art. Given the random nature of human motion, the promising frequency up-conversion (FUC) approach was applied to overcome the issues of PEHs related to their optimal performances at the respective eigenfrequencies only. Based on the initial studies available in literature, the influence of bimorph PEHs’ geometry on their outputs was systematically analysed and a DoE-based approach to geometry optimization was applied. Initial FE modal, harmonic and transient analyses allowed, then, establishing a clear indication of an increase in performances of the optimized bimorph PEHs as compared with the conventional one with a rectangular planar layout. Two optimized PEH designs (segmented and notched PEH) were, hence, proposed, both utilizing a larger portion of the available volume defined by the reference rectangular cantilever, compared with the solutions proposed in prior art.

By using the developed experimental setups, a thorough validation of the used numerical models was performed next, which proved the suitability of the used FE models. In fact, the measurement results, compared with FE data, prove not only the viability of the FE approach, but also the clearly improved performances of the optimized bimorph PEHs subject to excitation via plucking of their free ends, in terms of the normalised specific maximal output voltages and powers. In terms of the obtained specific maximal power outputs, a single trapezoidal segment of a three-segment device, displayed, thus, a 484% (in terms of the absolute maximal power: 28%) improvement in performance relative to the power output obtained via the reference rectangular bimorph, while the specific power output of the notched PEH increases by 86% (64.7% in terms of the maximal power). The addition of stress concentrators on the edges of the segments showed further improvements, i.e., an increase in specific power output of 14% (12% in maximal power) for the inverted trapezoidal shape, and a 32.5% (33.3% in maximal power) improvement for the trapezoidal segment. This, in turn, resulted in an increase in performances of 563% (43.5% when maximal power is considered), when a single trapezoidal segment with added stress concentrators is compared with the conventional rectangular bimorph. The inverted segments did display specific power-output levels that were slightly lower, although still comparable to the reference rectangular device, which can be attributed to the narrow cross-section near the clamped end that limited the allowable free-end plucking displacement. As repeatedly evidenced above, the latter power output should also be viewed as only a portion of the device’s overall power output that is to be added to that of the two trapezoidal segments, further confirming the viability of the segmented-design configuration approach.

The absolute power outputs potentially achievable by the optimized PEHs excited at the maximal allowable displacements of the free end, ranged, in turn and in terms of the maximal power outputs, from *P*_max_*δ*zmax_ = 9.26 mW, for the inverted trapezoidal PEH, to *P*_max_*δ*zmax_ = 134.06 mW, when the straight-edged segmented PEH is considered. In terms of the average power outputs, in the oscillation periods, characteristic of the considered shapes, the power outputs ranged, then, from *P*_ave_*δ*zmax_ = 0.22 mW, for the inverted trapezoidal PEH, to *P*_ave_*δ*zmax_ = 3.38 mW, for the straight-edged segmented bimorph PEH. The obtained overall performances of the straight-edged segmented design configuration are, therefore, significantly better than those of the conventional rectangular PEH bimorph with the same footprint (*P*_max_*δ*zmax_ = 34.8 mW and *P*_ave_*δ*zmax_ = 2.49 mW).

It should also be noted, here, that, when the results attained while keeping the excitation magnitude the same for all the PEH shapes, without considering stress limitations—as done in the initial studies and in most of the available literature—are compared to the results where the limitations caused by strength considerations are duly taken into account, a noteworthy difference in trends and performances is obtained. In fact, when the strength considerations are neglected, the inverted trapezoidal PEH clearly outperforms the trapezoidal one, but when the initial plucking deflection is limited due to excessive stress levels in the narrowest cross-section of the inverted shape, the trapezoidal bimorph shows much better performances. This demonstrates that the performances of the bimorph PEHs reported in literature, where strength considerations were neglected, should be considered with a fair degree of attentiveness.

The performed strength analyses then permit clearly establishing the allowable free-end-deflection and tip-mass values for each of the considered bimorph design configurations that still ensure a long and safe operation of the PEH transducers. The performances of the herein proposed PEHs, compared to a notable portion of the available literature, represent, therefore practically achievable values that can be expected for a long-lasting and optimally operating wearable devices used in real-life operating conditions. What is more, the proposed segmented PEH not only permits optimally using the available design volume—thus, amply meeting the power requirements needed for the wearable sensor nodes constituted by medical sensors with the respective data elaboration and communication modules as established in [3]; it also introduces the possibility of matching the response of each harvester segment with a different resistive load (i.e., sensor with the respective add-on modules), further improving the overall efficiency of the suggested wearable devices.

A kinetic EH approach, aimed at powering wearable devices and using an innovative combination of the FUC excitation modality with PEH geometry optimization, all with the goal of maximizing the resulting efficiency and energy density, along with a careful consideration of the dynamic strength and durability of the devices, are, therefore, all significant contributions of the herein performed work.

Given the influence of the initial free-end displacements on the outputs of the optimised harvesters, a thorough study of the influence of the stiffness (dependent also on the 3D printing parameters) and of the excitation frequency of the used plucking plectra on the FUC responses is under way, which will extend the herein presented treatise. From the design point of view, an improved fixture base is also being developed to facilitate a more precise clamping of the harvesters. In future work, additional harmonic excitation experiments will also be performed, along with those varying load resistances, so as to understand better the influence of the latter on the coupled electromechanical performances of bimorph PEH devices. A suitable power-management system is also being developed, which should enable matching different loads with each PEH segment, thus further optimizing the overall performances of the wearable devices. A detailed study of the damping effects is also planned.

All this will lead to the design of an integrated wearable device, comprising optimized bimorph PEHs excited by human and other random motion via the FUC approach, using a flywheel with several plectra, which will result in an autonomous apparatus that could be used in the nodes of sensor networks in telemedicine, in remote patient monitoring and in autonomous drug delivery. Such a device could also be implemented as a worker-safety monitor in dangerous working environments, in industry 4.0, in IoT settings and in structural health-monitoring (SHM) systems for e.g., airplanes or for civil engineering structures.

## Figures and Tables

**Table 1:** Properties of PZT-5A and stainless steel [39].

PZT-5A (3195HD)			
Elastic modulus	*E*_PZT_	52	GPa
Poisson ratio	*ν*_PZT_	0.31	-
Density	*ρ*_PZT_	7800	kg/m^3^
Piezoelectric strain coefficients	*d*_31_	390	pC/N
	*d*_33_	−190	pC/N
**Stainless Steel**			
Elastic modulus	*E*_s_	193	GPa
Poisson ratio	*ν*_s_	0.29	-
Density	*ρ*_s_	7800	kg/m^3^

**Table 2:** Comparison of optimized PEH parameters [38].

	R_L opt_, kΩ	P_max_, μW	P_Smax_, μW/m^2^
Trapezoidal	7	26.3	175.5
Inverted	12	131.5	672.7
Notched	7	168.9	545.9
Rectangular	5	141.3	409.5

**Table 3:** Mesh sensitivity validation for hexahedral (brick) elements with merged nodes.

Element Length, mm	*f*_1_, Hz	*e*_r_base_, %	*e*_r_FreeEnd_, %
2	503.32	6.91	6.13
1.75	532.69	1.25	0.46
1.5	540.11	0.14	0.93
1.25	544.05	0.86	1.65
1	525.13	2.68	1.89
0.75	534.79	0.85	0.06
0.5	534.28	0.95	0.16
0.25	539.45	0.01	0.80

**Table 4:** Mesh sensitivity analysis for hexahedral (brick) elements with glued volumes.

Element Length, mm	*f*_1_, Hz	*e*_r_base_, %	*e*_r_FreeEnd_, %
2	503.62	6,86	6.07
1.75	532.5	1.28	0.49
1.5	540.11	0.14	0.93
1.25	544.05	0.86	1.65
1	525.13	2.68	1.89
0.75	534.79	0.85	0.06
0.5	534.28	0.95	0.16
0.25	539.45	0.01	0.80

**Table 5:** Mesh sensitivity analysis for tetrahedral elements with glued volumes.

Element Length, mm	*f*_1_, Hz	*e*_r_base_, %	*e*_r_FreeEnd_, %
2	567.97	5.16	5.95
1.75	554.15	2.70	3.49
1.5	542.6	0.60	1.39
1.25	538.63	0.14	0.65
1	544.35	0.92	1.71
0.75	543.65	0.79	1.58
0.5	542.04	0.49	1.28
0.25	543.66	0.79	1.58

**Table 6:** Experimentally assessed mechanical eigenfrequency values compared with the modal FEA results.

	f_1_FEA_, Hz	f_1_EXP_, Hz	Diff.
Trapezoidal	930.2	934.1 (σ = ±1.63)	0.44%
Inverted	324.5	324.7 (σ = ±0.46)	0.065%
Notched	374.1	374.6 (σ = ±0.79)	0.14%
Trap. with wavy edges	762.5	793.7 (σ = ±2.98)	4.09%
Inv. with wavy edges	333.5	333.6 (σ = ±0.6)	0.033%
Rectangular	534.8	535.1 (σ = ±1.04)	0.056%

**Table 7:** Experimentally obtained voltage and power values.

	U_max_p-p_, V	P_av_, μW	P_max_, mW	P_s_max_, μW/mm^2^
Trapezoidal	11.46	71	2.79	37
Inverted	9.66	25	1.1	6
Notched	23.12	411	12.66	42
Trapezoidal with wavy edges	41.62	442	31.95	43
Inverted with wavy edges	17.9	148	4.74	24
Rectangular	20.1	468	12.42	36

**Table 8:** Experimentally obtained voltages and powers normalized by the respective *δ*_z_ values.

	U_n_max_p-p_, V/mm	P_n_ave_, μW/mm	P_n_max_, mW/mm	P_n_s_max_, μW/mm^2^/mm
Trapezoidal	45.83	1130	44.57	590
Inverted	36.46	360	15.43	80
Notched	49.18	2087	57.33	188
Trapezoidal with wavy edges	52.03	690	49.92	670
Inverted with wavy edges	37.3	641	20.57	106
Rectangular	33.4	2490	34.8	101

**Table 9:** Maximal and average power outputs achievable by the optimized PEHs at the maximal allowable initial free-end displacements *δ*_zmax_, as determined in Section 5.

	P_max_δzmax_, mW	P_ave_δzmax_, mW
Trapezoidal	62.4	1.58
Inverted	9.26	0.22
Notched	28.7	1.04
Trapezoidal with wavy edges	49.9	0.69
Inverted with wavy edges	12.3	0.38
Rectangular	34.8	2.49

## Data Availability

The following data files are available online at https://repository.riteh.uniri.hr/islandora/object/riteh:2274: Figure 3: ADL_accel_measurement, Figure 9: FEA_bending_stress_0.5mm, Figure 10: FEA_bending_stress, Figure 11: FEA_max_power_mass, Figure 12: EXP_log_decrement, Figure 13: thickness_meas, Table 3, Table 4 and Table 5: FEA_mesh_sensitivity, Figure 14: FEA_power_at_load, Figure 15, Figure 16, Figure 17, Figure 18 and Table 6: model_validation, Figure 19: EXP_voltage_FUC, Table 7, Table 8 and Figure 20: EXP_powers.
